# No Long-Term Effect of Physical Activity Intervention on Working Memory or Arithmetic in Preadolescents

**DOI:** 10.3389/fpsyg.2017.01342

**Published:** 2017-08-10

**Authors:** Douglas Sjöwall, Mattias Hertz, Torkel Klingberg

**Affiliations:** ^1^Department of Neuroscience, Karolinska Institute Stockholm, Sweden; ^2^Enköping Municipality Uppsala, Sweden

**Keywords:** physical exercise, cognition, intervention, chronic, arithmetic, grit, working memory

## Abstract

We investigate if increased physical activity (PA) leads to enhanced working memory capacity and arithmetic performance, in a 2-year school-based intervention in preadolescent children (age 6–13). The active school (*n* = 228) increased PA (aimed at increasing cardiovascular fitness) from 2 to 5 days a week while the control school (*n* = 242) remained at 2 days. Twice a year, participants performed tests of arithmetic as well as verbal and spatial working memory. They also rated stress with a questionnaire at the start and at the end of the intervention. There was no beneficial development of working memory or arithmetic for the active school as compared to the control school. Furthermore, subgroup analyses revealed no favorable intervention effect for high/low baseline fitness, cognition or grit. Unexpectedly, a significant increase in self-rated stress was detected for the active school and this effect was driven by girls rather than boys and by the younger rather than older children. These results indicate that longtime high intensity PA does not lead to a beneficial development of working memory or arithmetic in preadolescent children.

## Introduction

Several studies have shown a positive correlation between physical fitness and working memory (Raine et al., 2016) as well as arithmetic (Donnelly et al., 2016). Also, a longitudinal study has shown that gain in fitness is associated with gain in working memory (Scudder et al., 2016). However, a number of meta-analyses have given a more nuanced picture of this relation (Verburgh et al., 2014; Cooper et al., 2016; Spruit et al., 2016), pointing out that some of the positive correlations are from cross-sectional studies where there is a risk of confounding variables, such as genetics or general health, explaining the association.

Less data is available for controlled interventions, especially in preadolescents. Moreover, as several forms of physical activity (PA) interventions exist, varying in length (i.e., acute/chronic) and intensity (e.g., motor skills/aerobic), there is a need to be specific about what type of intervention affects what specific ability if we are to make more accurate inferences of the effect of PA on cognition. Additional information is needed before making policy decisions such as a mandatory increase in PA for children.

Here, we have reviewed the literature and investigated the effect of PA on functions that are highly relevant for academic achievement: working memory and arithmetic, in a 2-year school-based intervention in preadolescent children (age 6–13).

### Working Memory and PA

Working memory refers to the ability to retain information in memory over a short period of time (Conway et al., 2003) and is strongly associated with academic achievement (Bull et al., 2008). Deficits in working memory are common in neurodevelopmental disorders such as Attention Deficit Hyperactivity Disorder (Martinussen et al., 2005). Therefore, working memory is a viable candidate to target in interventions. Cross-sectional studies of 9–10 year-olds, has demonstrated an association between measures of physical fitness and working memory capacity (e.g., Raine et al., 2016) but less is known regarding a potential causal effect where increased PA would lead to improved working memory.

A randomized, controlled study found a positive effect of a 9-month (5 × 70 min per week) intervention mixing cardio, muscle and motor skills activities on working memory in 7–9 year-olds (Kamijo et al., 2011). However, the sample size was small (*n* = 43) and the authors raise the possibility that effects could be due to a regression toward the mean-effect. Also, a more recent study including 9–10 year-olds did not replicate this finding for 10 weeks (3 × 45 min per week) of cardiovascular training (Koutsandreou et al., 2016). Finally, as findings of a recent study suggested that cardiorespiratory fitness is selectively related to better working memory performance for boys rather than girls (Drollette et al., 2016), it will there fore be important to investigate if PA have different effects on boys and girls over time.

### Mathematics and PA

Poor mathematics achievements are associated with academic underperformance in general and are also associated with poor future economical and health outcomes (Butterworth et al., 2011). Regarding the possible association between PA and mathematics, a recent review concluded that results are mixed and there is a need for well-designed studies (Donnelly et al., 2016). However, based on the longitudinal studies mentioned in this review, there are indications of a beneficial effect of a 9-month (3 × 30 min per week) aerobic dance intervention in a repeated-measures crossover design on mathematics in grades 3–5 (Gao et al., 2013) and a physical exercise intervention throughout grades 1–6 (Shephard, 1996). However, the latter (Shephard, 1996) found beneficial effects for mathematics in only 4 out of 12 comparisons and it can therefore be questioned to what extent this should be considered a beneficial effect.

In a recent study, the effect of a 7-month PA intervention (including 90 min per week of physically active lessons, 5 min per day of PA breaks and an additional 10 min per day of PA homework) on academic development was investigated in 1129 fifth-grade children with a cluster-randomized controlled trial (Resaland et al., 2016). No significant treatment effects were seen for mathematics. However, a subgroup analysis revealed a beneficial effect for those who performed the poorest in mathematics at baseline. Although this intervention combined several types of PA and educative modalities, which therefore makes the active component hard to isolate, it showed the need for conducting subgroup analyses. Also, a recent study showed that PA was differently related to arithmetic in boys than in girls (Haapala et al., 2017). Thus, it will be import to test whether girls and boys are affected differently by PA using a controlled trial design.

### Stress as a Mediating Effect of PA

Some of the physiological effects of PA include increased levels of brain-derived neurotrophic factor (BDNF), which in turn could affect neuronal plasticity and cognitive functions (e.g., Ferris et al., 2007). PA can also have beneficial effects on objective measures of stress such as lowered blood pressure (Janssen and LeBlanc, 2010). Both chronic and acute stress can impair cognitive functions, including working memory (Leach and Griffith, 2008; Evans and Schamberg, 2009) and arithmetic (Schmader and Johns, 2003). In the present study we evaluated long-term effects of PA on stress. Our hypothesis was that PA would reduce stress, and that this effect in turn could partially mediate a beneficial effect of PA on working memory and arithmetic.

### Grit and PA

Grit is a personality trait that quantifies a person’s ability to persist with an activity despite setbacks, and to pursue long-term goals (Duckworth et al., 2007). It has typically been estimated through self-rating according to a 12-item questionnaire.

This trait has been shown to predict drop-out rates in college as well as spelling progress (Duckworth et al., 2007; Duckworth and Quinn, 2009; Eskreis-Winkler et al., 2014). More recently, grit was shown to predict training progress in 6-year old children doing working memory training (Nemmi et al., 2016). As grit has been shown to predict amount of progress during training, we included it here to investigate if it could interact with the training effect, with the hypothesis that children in the active school with high grit would benefit more than those with low grit.

### Aim

To conclude, few controlled longitudinal studies have been conducted and there is limited knowledge regarding the effects of PA on children’s working memory and arithmetic capacity. The aim was therefore to investigate if increased long-term PA leads to enhanced working memory capacity and arithmetic performance.

## Materials and Methods

### Design and Participants

The study is based on data from a 2-year school-based intervention including two schools: one active school with increased PA *n* = 228 (55% boys) and one control school *n* = 242 (48% boys). The schools were located in the same area outside Enköping, Sweden and were chosen based on their similarity on socio economic characteristics. Pupils who attended the schools were in 1st to 6th grade (6–13 years old). Children who were in grade 1–5 at the start of the study were included during 2 years. During the second year of the study new grade 1 subjects were included, while those who were in 6th grade during first year were not included when they entered 7th grade. This intervention was initiated and funded by Enköpings municipality. The first and the senior author were recruited to provide a non-biased scientific evaluation after completing 2 years of the intervention. An ethical application was submitted to the regional ethics committee in Stockholm who found no ethical objection and stated that the projected did not require an ethical approval. Data were anonymized before analysis.

### The Intervention

Both schools had 120 min (2 × 60 min) of curriculum-prescribed PA per week as part of their mandatory curriculum. Based on previous studies showing an association between PA and cognition (Raine et al., 2016) and academic performance (Donnelly et al., 2016) the active school established an additional 180 min (3 × 60 min). For the additional PA in the active school, about 20 of the 60 min of PA consisted of showering and change of clothing (i.e., 120 min of active PA per week). In the active school, PA was spread out so that pupils were active every day. The active school employed an activity leader who was responsible for organizing the daily PA. PA classes were high intensity based on the results of large study showing a positive association between cardiovascular fitness and cognition in early adulthood (Aberg et al., 2009). Activities included in the intervention required no previous knowledge or skills. The PA was varied so that it would be fun for the pupils and consisted of aerobics classes, obstacle course, boxing, skipping rope, running, and various forms of high-intensity play. PA was mandatory and adherence to the PA was high.

### Outcome Measures

Pupils in both the active and in the control school were assessed four times during a 2-year period: once in the beginning of each fall and in the end of each spring. The participants who attended the 6th grade when the study started were only present for two measurements as they left the school 1 year into the intervention. The same is true for children who started their first school year 1 year into the intervention. The primary outcome measures were two working memory tasks and an arithmetic (images of the tasks is found in the Supplementary Material) test previously used to evaluate the effect of working memory interventions in the same age-group (Bergman-Nutley and Klingberg, 2014). Also, measures of stress, grit and physical performance were included in order to provide more elaborate analyses of development. The following variables were included in the study:

#### Working Memory

Working memory was measured using a spatial and a verbal task. Both were performed on a computer. The spatial task was the Odd One Out task based on the Automated Working Memory Assessment (Alloway, 2007). In this task, participants are instructed to identify which shape out of three is the odd one and remember its location. The shapes are present until the participant responds. This is repeated a number of times based on level. Participants are then presented with three empty slots and should indicate the locations where the odd shapes appeared, in the order they appeared. The test stops when the participant answers incorrectly for two trials on the same level. The highest level where at least one trial was correct is used to calculate the performance on this task. The same rules for progression, stopping and scoring are used in the verbal working memory task described below. The correlations between the four consecutive time points were in the range 0.51–0.57, *p*s < 0.001.

In the verbal working memory task, participants are asked to follow progressively longer instructions (Gathercole et al., 2008; Bergman-Nutley and Klingberg, 2014). Common classroom items are placed on a table (e.g., eraser, pencil, and box) and the task is to follow the verbal instructions (e.g., “Click on the pencil, then drag the eraser to the box”). This example would be a trial on level three (there are three items to remember what to do with). After practice trails, participants start on level two. The correlations between the four consecutive time points were in the range 0.52–0.56, *p*s < 0.001.

#### Arithmetic Test

In this test participants were asked to solve as many mental arithmetic problems (addition and subtraction) as possible during 1 min. The tasks included two or three terms with a sum less than 20 and did not include duplicate terms or numbers with zero in them. For more detailed information regarding the two working memory and arithmetic task, please see Bergman-Nutley and Klingberg (2014). The correlations between the four consecutive time points were in the range 0.77–0.84, *p*s < 0.001.

#### Physical Performance

For the purpose of assessing physical ability, participants conducted a “Beep test” and ran 1700 m. For the 1700 m test, the time it took for participants to complete the course was used as the outcome measure (i.e., lower scores indicate a better performance). The correlations between the consecutive time points were in the range 0.68–0.74, *p*s < 0.001.

In the Beep test (also known as “Shuttle run”), participants run side to side between two points that are 20 m apart. The laps (a run between the two points) are synchronized by a beep. After a completed level of laps, the interval between the beep decreases so that participants have to run faster to the point when they cannot run the lap in time. For exact speed and number of laps within each level please see Leger et al., 1988. In contrast to the 1700-m test, the entire performance was supervised by a teacher. For the Beep test, completed laps and levels were converted to meter that were used as the outcome measure. At the first measurement, participants in the control school accidently ran intervals of 18 instead of 20 meters, but as the analyses were calculated in terms of meters, this should have no or minimal impact. Also, as removing measurement one did not change the slope of development, results are presented using data for all four measurement points. The correlations between the consecutive time points were in the range 0.66–0.82, *p*s < 0.001.

#### Stress

Stress was measured with a questionnaire filled out by the child together with their parents. The questionnaire includes 11 items rated on a 5-point scale from 1 = “never” to 5 = “always.” A previous factor analysis yielded two factors: *pressure* (e.g., “I rush even if I don’t have to”) and *activation* (e.g., “I do not have time enough”) which were shown to have satisfactory internal consistency (Lindblad et al., 2008). Questions regarding stress were measures twice; in the beginning and in the end of the intervention corresponding to when the working memory and arithmetic were measures the first and last time. The correlation between the two time points was 0.56, *p* < 0.001.

#### Grit

Grit was measured at baseline using a 12-item grit-scale (Duckworth and Quinn, 2009) completed by the child’s teacher and parent. The grit-scale used was previously translated into Swedish and made more suitable for younger children and was shown to have adequate psychometric properties (Nemmi et al., 2016).

### Statistical Analyses

First, outliers were handled using the outlier labeling rule (Hoaglin and Iglewicz, 1987) adjusting outliers to the upper or lower value via the formula (1st/3rd Quartile ± (2.2 × (3rd – 1st Quartile) for each group. Zero values in working memory or arithmetic tasks were excluded as it indicated that the participant did not try or understand the task. The pattern of results of the main analyses was the same both with and without the outliers and zero-scores. However, all analyses presented in the article excluded outliers and zero-scores. The missing data for each time point were in the range 6–15% for working memory and arithmetic, 28–35% for stress, 15–23% for the beep test, 15–27% for 1700-m, 8% for teacher rated grit, and 27% for parent rated grit. For the main analyses we used mixed models that is generally considered a good method for analyzing data when some data points are missing (e.g., if measurement for one out of four time points are missing, the participant is not excluded and the model makes use of the three existing time points). To be sure however, we also re-analyzed data for the main effects using multiple imputation and the pattern of results did not change.

Second, in order to investigate associations between fitness and working memory/arithmetic, cross-sectional correlations were calculated as well as regression analyses where **Δ** working memory/arithmetic were regressed on **Δ** fitness, baseline fitness, baseline working memory/arithmetic, age and gender. **Δ** fitness and working memory/arithmetic were calculated as the development between the first and the last measurement for these variables.

Third, the development of the schools was investigated for each outcome using mixed models procedures. For each outcome measure the effect of the school (dummy coded as 1 = active school and 0 = control school), time (1, 2, 3, 4), sex, and age (months) was calculated. The contrast of interest was thus the interaction of school × time, including all available time-points. This analysis gives the interaction between school and time while controlling for age and sex.

Forth, when investigating if high and low baseline physical performance affected the impact of the intervention, we used mixed models procedures. High and low was defined as performing above or below the 50 percentile at baseline 1700-m or the Beep test for each school respectively. The scores were age- and sex-adjusted using standard regression procedures. We also investigated the impact of high and low baseline working memory/arithmetic on the intervention for each outcome. Following the same logic, the effect of high and low grit at baseline was investigated for the three-way interaction of school × time × high/low.

Finally, the three-way interaction of school × time × age as well as school × time × sex was investigated for the development of working memory, arithmetic and stress. Effect sizes were calculated using Cohen’s delta (d) where 0.2 is considered a small effect, 0.5 a medium effect, and 0.8 a large effect (Cohen, 1988).

## Results

### Baseline Analyses

Within age means and standard deviations were calculated for the outcome variables (see **Table 1**). There were significant associations between baseline physical performance as measured with the Beep test in relation to baseline performance in the Odd One Out (*r* = 0.32, *n* = 317, *p* < 0.001), the follow instructions task (*r* = 0.21, *n* = 367, *p* < 0.001), and arithmetic (*r* = 0.34, *n* = 333, *p* < 0.001). The associations for the Odd One Out (*r* = 0.15, *n* = 313, *p* < 0.01) and the arithmetic task (*r* = 0.14, *n* = 330, *p* < 0.05) remained significant when controlling for age but not for the follow instructions task (*r* = 0.01, *n* = 364, *ns*). Similarly, there were significant associations between baseline physical performance as measured with the 1700 m test in relation to baseline performance in the Odd One Out (*r* = -0.19, *n* = 317, *p* < 0.001), follow instructions task (*r* = -0.20, *n* = 367, *p* < 0.001), and arithmetic (*r* = -0.35, *n* = 333, *p* < 0.001). The association to arithmetic remained significant when controlling for age (*r* = -0.17, *n* = 330, *p* < 0.01) whereas associations to the Odd One Out (*r* = -0.03, *n* = 314, *ns*) and the follow instructions task (*r* = -0.03, *n* = 364, *ns*) did not.

**Table 1:** Mean (*M*) and standard deviations (*SD*) for outcome variables at baseline for grades 1–6.

	Age group 1 *M* (*SD*)	Age group 2 *M* (*SD*)	Age group 3 *M* (*SD*)	Age group 4 *M* (*SD*)	Age group 5 *M* (*SD*)	Age group 6 *M* (*SD*)
Odd One Out	2.76 (1.01)	2.78 (1.05)	4.16 (1.16)	3.77 (1.19)	4.05 (1.02)	4.38 (1.21)
Follow instructions	2.36 (0.82)	2.49 (0.81)	3.45 (0.87)	3.12 (0.92)	3.39 (0.89)	3.65 (0.73)
Arithmetic	1.91 (1.73)	2.99 (2.71)	8.97 (3.98)	8.05 (4.10)	9.82 (4.75)	9.57 (3.99)
Stress	1.75 (0.51)	1.92 (0.47)	1.89 (0.54)	1.94 (0.62)	2.04 (0.48)	2.12 (0.66)


Furthermore, when comparing change in fitness over 2 years (irrespective of interventions) larger improvements in the Beep test were positively related to larger changes in the Odd One Out, β = 0.17, *p* < 0.01. There was a trend toward significance for larger changes in the follow instructions task, (β = 0.10, *p* = 0.06), but not for arithmetic, (β = -0.02, *p* = 0.827). Larger improvement for 1700 m were not significantly associated with larger changes for the Odd One Out, β = -0.08, *p* = 0.142, or for the follow instructions task, β = -0.05, *p* = 0.39, but there was a trend toward a significant effect for arithmetic, β = -0.12, *p* = 0.08.

The correlation between the beep test and 1700 m was in the range 0.59–0.71, *p*s < 0.001, at each time point. The correlation between the Odd One Out task and the follow instructions task was in the range 0.40–0.46, *p*s < 0.001, at each time point.

### Main Effects of the Intervention for Fitness Measures

There was a significant time × school interaction [*F*(1,956) = 88.22; *p* < 0.001; *d* = 0.64] for the Beep test thus indicating that the development for the schools differed over time. As expected, the control school remained on a steady level while the active school improved almost 50%. Regarding 1700-m performance, the was a significant time × school interaction [*F*(1,906) = 36.79; *p* < 0.001; *d* = 0.58] thus indicating that the development for the schools differed over time. However, in contrast to what was expected, the decrease in time it took to finish the 1700-m course was larger for the control school. This result was also confirmed using an alternative analysis with multiple imputations [*F*(1,1048) = 26.74; *p* < 0.001; *d* = 0.28].

### Main Effects of the Intervention for Working Memory/Arithmetic/Stress

**FIGURE 1:**
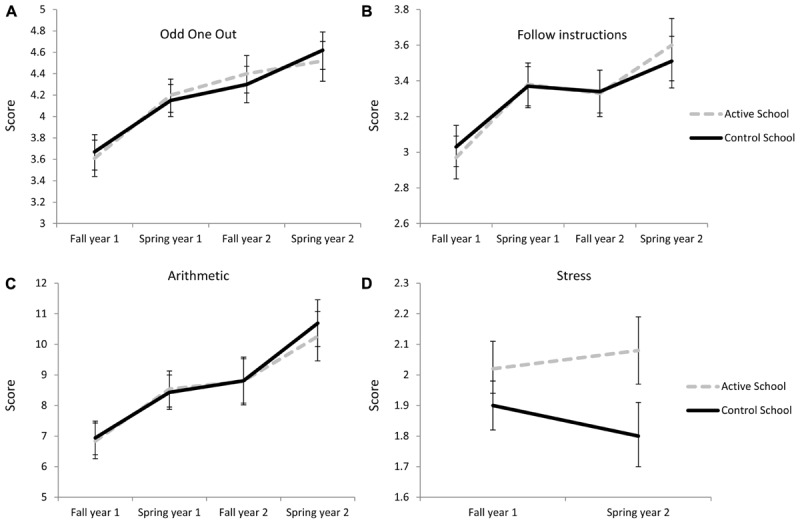
Development, controlled for age and sex, for the active and the control school over 2 years measured in the beginning of each fall and in the end of each spring for **(A)** spatial working memory, **(B)** verbal working memory, **(C)** arithmetic. Development of stress **(D)** was measures twice, once in the beginning and once in the end of the 2-year period. Error bars are based on the 95% confidence interval.

No significant time × school interactions were found for the Odd One Out task [*F*(1,1062) = 0.05; *p* = 0.83; *d* = 0.01], follow instructions task [*F*(1,1126) = 1.21; *p* = 0.27; *d* = 0.05], or for the arithmetic task [*F*(1,1021) = 0.42; *p* = 0.52; *d* = 0.11]. As can be seen in **Figures 1A–C**, the two schools follow each other over time and there is no indication that the increased amount of PA carried out in the active school had any effect on working memory or arithmetic.

For stress however, a significant time × school interaction was found [*F*(1,793) = 20.69; *p* < 0.001; *d* = 0.38; **Figure 1D**]. The two schools develop in opposite direction with the control school slightly decreasing while the active school slightly increases in stress levels.

### Treatment Effects in Relation to Baseline Physical Performance

No significant time × school × baseline physical performance (Beep test) interactions were found for the Odd One Out task [*F*(1,923) = 0.61; *p* = 0.44], the follow instructions task [*F*(1,979) = 1.23; *p* = 0.27] or for stress [*F*(1,705) = 0.11; *p* = 0.74]. The lack of a three-way interaction indicates that the school × time two-way interaction does not differ between children with high or low baseline physical performance.

However, there was a significant time × school × baseline physical performance interaction as measured with the Beep test for arithmetic [*F*(1,885) = 12.70; *p* < 0.001]. In order to explore these associations the main analyses (time × school) were re-run separately for low and high baseline physical performance groups. Significant interactions effects were seen in both the low baseline group and in the high baseline group. In the low baseline group, the largest increase was seen for the active school [*F*(1,444) = 4.77; *p* < 0.05; *d* = 0.32]. Unexpectedly however, the largest increase in the high baseline group was observed for the children in the control group [*F*(1,446) = 7.76; *p* < 0.01; *d* = 0.42]. No significant interaction effects were found when the same analyses were performed with 1700-m performance instead of the beep test (all *F*s < 2.21).

### Treatment Effects in Relation to Baseline Working Memory and Arithmetic Performance

**FIGURE 2:**
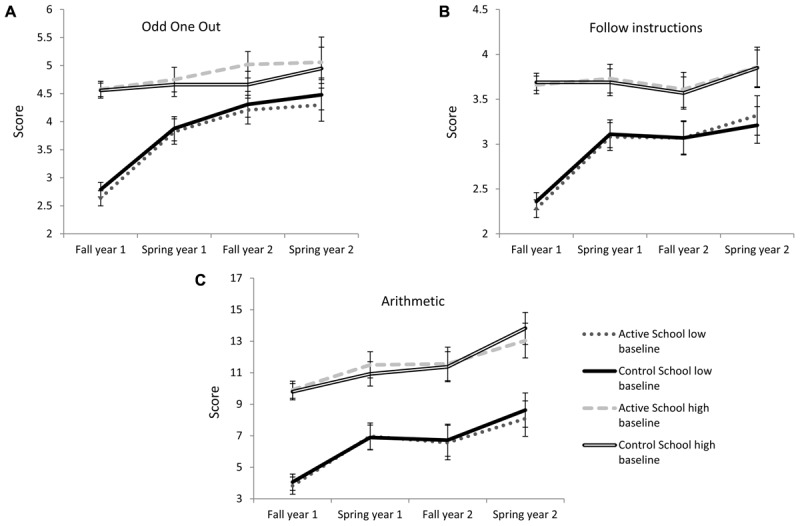
Development, controlled for age and gender, for the active and the control school over 2 years measured in the beginning of each fall and in the end of each spring for **(A)** spatial working memory development separated by baseline performance in spatial working memory, **(B)** verbal working memory development separated by baseline performance in verbal working memory, **(C)** arithmetic development separated by baseline performance arithmetic. Error bars are based on the 95% confidence interval.

Next, we investigated if high and low baseline working memory/arithmetic performance affected the impact of the intervention using mixed models. No significant time × school × baseline working memory/arithmetic performance interactions were found in relation to their respective development (all *F*s < 0.85) indicating that development does not differ between children with high or low baseline performance between the different schools (see **Figures 2A–C**).

### Treatment Effects in Relation to Baseline Grit

There were no significant time × school × baseline grit interactions for working memory, arithmetic or stress as assessed by either parents or teachers (all *F*s < 2,23). This indicates that baseline grit does not change the effect of the intervention.

### Treatment Effects in Relation to Age

No significant time × school × age interactions were found for the Odd One Out task [*F*(1,1330) = 0.21; *p* = 0.65], or for arithmetic [*F*(1,1333) = 0.74; *p* = 0.39]. The lack of a three-way interaction indicates that the school × time two-way interaction does not differ between children with regard to age. However, there was a significant time × school × age interaction for the follow instructions task [*F*(1,1420) = 4.10; *p* < 0.05] and for stress [*F*(1,922) = 5.41; *p* < 0.05; see Supplementary Material]. In order to explore these associations the main analyses (time × school) were re-run separately for the younger and the older half of the sample. For the follow instructions task, no significant time × school interaction were found in either the younger or the older half of the sample (*F*s < 1.53; *d*s < 0.13). For stress, there were no interaction in the older half [*F*(1,405) = 0.52; *p* = 0.47; *d* = 0.22], but a significant interaction in the younger half [*F*(1,383) = 41.66; *p* < 0.001; *d* = 0.60] indicating that younger children in the active school increased their stress more than the controls. We also investigated non-linear effects using age^2^ but we did not find such effects.

### Treatment Effects in Relation to Sex Differences

Finally, significant time × school × sex interactions were found for the follow instructions task [*F*(1,1415) = 3.92; *p* < 0.05], arithmetic [*F*(1,1322) = 6.73; *p* < 0.01], stress [*F*(1,946) = 5.22; *p* < 0.05] and a trend toward a significant effect for the Odd One Out task [*F*(1,1319) = 3.68; *p* = 0.055]. In order to explore these associations, the main analyses (time × school) were re-run separately for boys and girls. Regarding the follow instructions task, no significant differences were found for boys [*F*(1,586) = 0.54; *p* = 0.81; *d* = 0.05] whereas girls in the active school increased more than girls in the control school [*F*(1,536) = 4.57; *p* < 0.05; *d* = 0.20]. The same pattern was seen for stress [*F*(1,406) = 30.55; *p* < 0.0001; *d* = 0.46] indicating that girls in the active school increased their stress levels more than girls in the control school. As we also found a time × school × age in relation to stress indicating that younger children in the active school increased their stress more than the controls, time × school were re-run for young boys and girls separately. However, significant interactions effects were seen for both boys [*F*(1,177) = 16.44; *p* < 0.001; *d* = 0.60] and girls [*F*(1,203) = 23.57; *p* < 0.001; *d* = 0.65]. No significant treatment effects were found for the Odd One Out or arithmetic in either boys or girls (*F*s < 2.85).

## Discussion

In this study, a school-based, 2-year intervention of PA was evaluated for it’s effect on working memory and arithmetic capacity. Baseline physical fitness correlated with performance on tests of working memory and arithmetic and there were some indication of that gain in physical improvement was related to gain in these variables.

However, when the active school was compared to the passive school over 2–years time, no significant effects were found for working memory or arithmetic. A large number of subgroup analyses were performed. For one out two working memory measures, an interaction effect was found indicating that girls had larger treatment effects. Also, children in the lower half on physical performance at baseline increased more in arithmetic than controls. However, these effects did not survive correction for multiple comparisons. Importantly, this study shows that although there are associations between fitness and working memory and arithmetic, increasing PA does not seem to generate higher working memory or arithmetic capacity. Moreover, contrary to our hypothesis, an increase in stress was observed for the active school. Further analyses showed that this effect was driven by girls rather than boys and by the younger rather than older children.

### Working Memory

Similar to Koutsandreou et al. (2016) who also studied the effects of a high intensity PA intervention, we did not find any effect of PA on working memory. Importantly, in the present study this was corroborated using a larger sample and an intervention that lasted over a much larger time period.

As a previous study found that subgroups responded differently to the intervention (Resaland et al., 2016), we investigated development based on subgroups for baseline grit, working memory/arithmetic and physical performance. However, we found no support for increase in PA with improvement of working memory. Although Koutsandreou et al. (2016) did not find an effect of cardiovascular PA, another group who trained motor skills exercises did improve working memory significantly more than controls. Similarly, this was also observed in another study including elements of motor skills (Kamijo et al., 2011).

As the sample in the present study included a wide age range, we tested if the effect of the intervention differed with age. Treatment effects did not differ depending on the age of the children for spatial working memory task but there was a significant interaction effect for verbal working memory. As the intervention lasted for 2 years and because children thus moved within the age range included, it is hard to isolate age groups that represent the model used to detect the interaction effect. However trying to explore this effect further by splitting the sample based on age at baseline, we were not able to see that the intervention was differently effective when comparing active children to same aged controls. Moreover this effect did not survive when correcting for multiple compassions and the effect was only seen in one of the two working memory variables and not in arithmetic. Therefore, it will be important to investigate if children are more susceptible to PA at any particular age in future studies.

Based on previous findings suggesting that the association between physical fitness and working memory is stronger in boys than in girls (Drollette et al., 2016), we also investigated if the intervention affected girls and boys differently. Unexpectantly, we found an interaction effect suggesting that girls in the active school were the ones that improved the most in working memory. However, this effect was only seen in one of the two working memory variables and the effect did not survive when correcting for multiple comparison. Moreover, the magnitude of the effect was small and seen in the context of deciding whether or not to introduce more PA for girls, this effect needs to be replicated.

### Arithmetic

No main effects of the intervention were found for arithmetic. This is in line with the study by Resaland et al. (2016), which included 1129 children in a 7-month, randomized, controlled trial. In that study, several academic subjects were included and they were able to show that compared to the control group there was no significant effect for numeracy, reading or English. However, beneficial effects of a PA intervention from a 9-month game-based dancing exercise (90 min per week), was shown by Gao et al. (2013). Although this intervention included high intensity PA it also included motor skills and arguably a cognitive load, as participants had to carry out the increasingly difficult instructions they saw on the screen. Thus, it is not clear if it is the PA, the cognitive load, motor skills or the combination of these modalities that caused the effect.

In contrast to Resaland et al. (2016) there was no favorable intervention effect for those who performed poorest at baseline in arithmetic in the present study. Although interventions had the element of PA in common, the children in that study underwent physically active lessons where they practiced simultaneous problem solving. It is therefore possible that improvements seen in relation to this intervention could be explained by this novel method for learning rather than through increased physical fitness.

However, there was some indication that low fitness children in the active school increased more in arithmetic than controls. This effect did not survive correction for multiple comparisons and was only seen for one out of two fitness measures. Furthermore, we did not find any indication that age changed the main results i.e., no effects of the intervention were found for arithmetic development.

### Stress

Unexpectedly, this study found that children in the active school experienced a slight increase in stress compared to the control school. Further analyses showed that this effect was driven by girls rather than boys and by the younger rather than older children. Although this result calls for more studies addressing the possible negative consequences of increased PA, it should be noted that the average stress level is located around 2 on a scale reaching from 1 to 5, indicating that they rarely experience stress. The results of this study also indicate that girls also seem to benefit the most from the intervention in terms of working memory development.

However, in the context of previous studies that suggest beneficial effects of PA on stress with objective and blinded measures (e.g., Janssen and LeBlanc, 2010) the finding in this study, which is assessed by children and their parents, should be interpreted with some caution. In the context of recommendations for more mandatory PA, it is worth noting that such increase in PA, although it probably has no negative effects on children’s health, comes with a price in terms of the time required by children, which could lead to increased stress.

### Limitations and Future Studies

This study has many methodological merits such as including a large sample, a controlled longitudinal design spanning over 2 years, specific and objective measures of working memory, arithmetic and physical ability. However, some limitations should be considered. This study did not include measures of height and weight although it has been shown in previous studies that such characteristics could be an important factor to study (Kamijo et al., 2012; Morita et al., 2016). There was no randomization as to who was in the active or the control group and we do not have access to demographic data. However, schools are located in similar rural areas within the same vicinity and were very similar in baseline working memory/arithmetic functioning. Furthermore, there we quite large amounts of missing data for some of the variables. However, the analyses are based on a much larger sample than what is common in previous studies within this field and analyses were performed using mixed models procedures that allow missing data.

Although the active school had a larger increase in physical ability as measured with the Beep test, children in the control school had a larger increase in physical ability as measured with the 1700 meters test (although the active school improved as well). It is possible that children at this young age do not maximize their performance in a long-distance test to the same extent as they do in the beep test. Performing the 1700-m test children run more on their own, without supervision, and they do not perhaps have a good sense of how fast they should run to perform at their best. In contrast, when performing the beep test, this is done in front of their peers and they also have the “beeps” to indicate how fast they should be running. However, as the active school had more than twice the amount of PA as the control school (approximately 1 h every school day for 2 years), it is highly likely that they benefitted physically from their training. Although it should be taken into account that the lack of beneficial effects from PA on working memory/arithmetic might be that children show a smaller trainability in terms of their oxygen uptake (VO2) peak as compared to adults (Matos and Winsley, 2007). Thus, it might be harder to produce a positive development of cognition via PA in prepubertal children.

Furthermore, it cannot be ruled out that PA has a positive effect on working memory and arithmetic but that this affect asymptote within the 2 h of training that the control school also performs, i.e., that the positive effect plateaus at 2 h of PA per week.

Nevertheless, the results of this study ad valuable information by showing that more than 2 h of training will not make a difference. It should also be noted that the negative findings in this study does not exclude effects for other cognitive abilities not measured here. It is also possible that intervention could lead to positive health effects such as cardio metabolic health later in life (Cooper et al., 2016).

## Conclusion

This study shows that increasing the amount of PA from 2 to 5 h per week, in preadolescent children, over 2-years did not affect working memory or arithmetic development and increased stress in girls and in younger children. This warrants some caution for introducing more PA in the curriculum when increased PA takes time from classes or homework.

## Author Contributions

Conceptualization: MH and TK; Data curation: MH and DS; Formal analysis: DS; Investigation: MH; Writing – original draft preparation: DS and TK; Writing – review and editing: DS, TK, and MH.

## Conflict of Interest Statement

Co-author MH is employed by Enköpings municipality. The other authors declare that the research was conducted in the absence of any commercial or financial relationships that could be construed as a potential conflict of interest.
