# Supplementary material for: No Long-Term Effect of Physical Activity Intervention on Working Memory or Arithmetic in Preadolescents
Source: Front Psychol. 2017 Aug 10;8:1342. doi: 10.3389/fpsyg.2017.01342 (PMC5554341; doi:10.3389/fpsyg.2017.01342)
Supplement: Supplementary file 1 [file Presentation_1.pptx]

## Slide 1
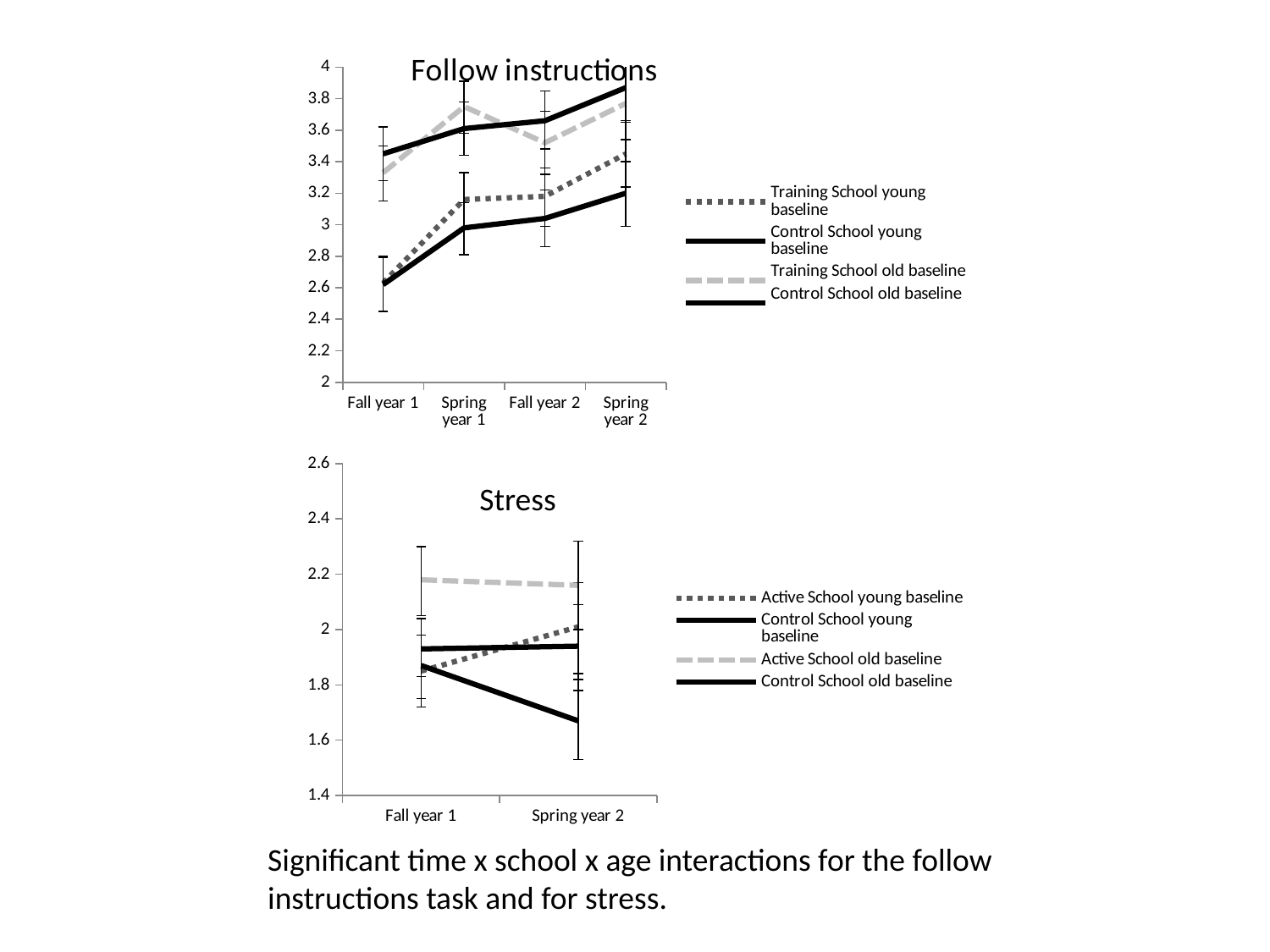

### Chart
| Category | Training School young baseline | Control School young baseline | Training School old baseline | Control School old baseline |
|---|---|---|---|---|
| Fall year 1 | 2.63 | 2.62 | 3.33 | 3.45 |
| Spring year 1 | 3.16 | 2.98 | 3.75 | 3.61 |
| Fall year 2 | 3.18 | 3.04 | 3.52 | 3.66 |
| Spring year 2 | 3.45 | 3.2 | 3.77 | 3.87 |
### Chart
| Category | Active School young baseline | Control School young baseline | Active School old baseline | Control School old baseline |
|---|---|---|---|---|
| Fall year 1 | 1.85 | 1.87 | 2.18 | 1.93 |
| Spring year 2 | 2.01 | 1.67 | 2.16 | 1.94 |Stress
Significant time x school x age interactions for the follow
instructions task and for stress.

## Slide 2
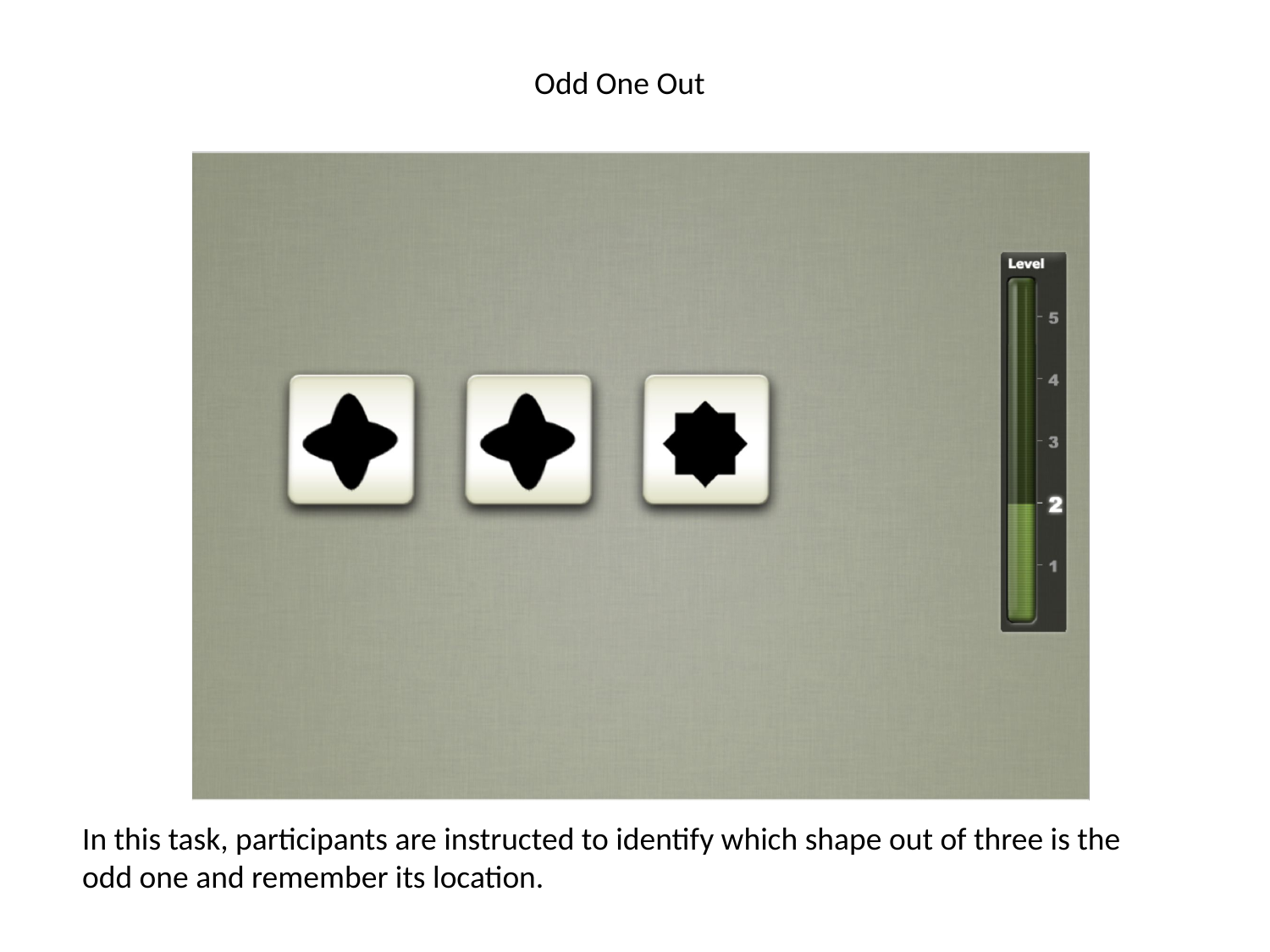

Odd One Out
In this task, participants are instructed to identify which shape out of three is the odd one and remember its location.

## Slide 3
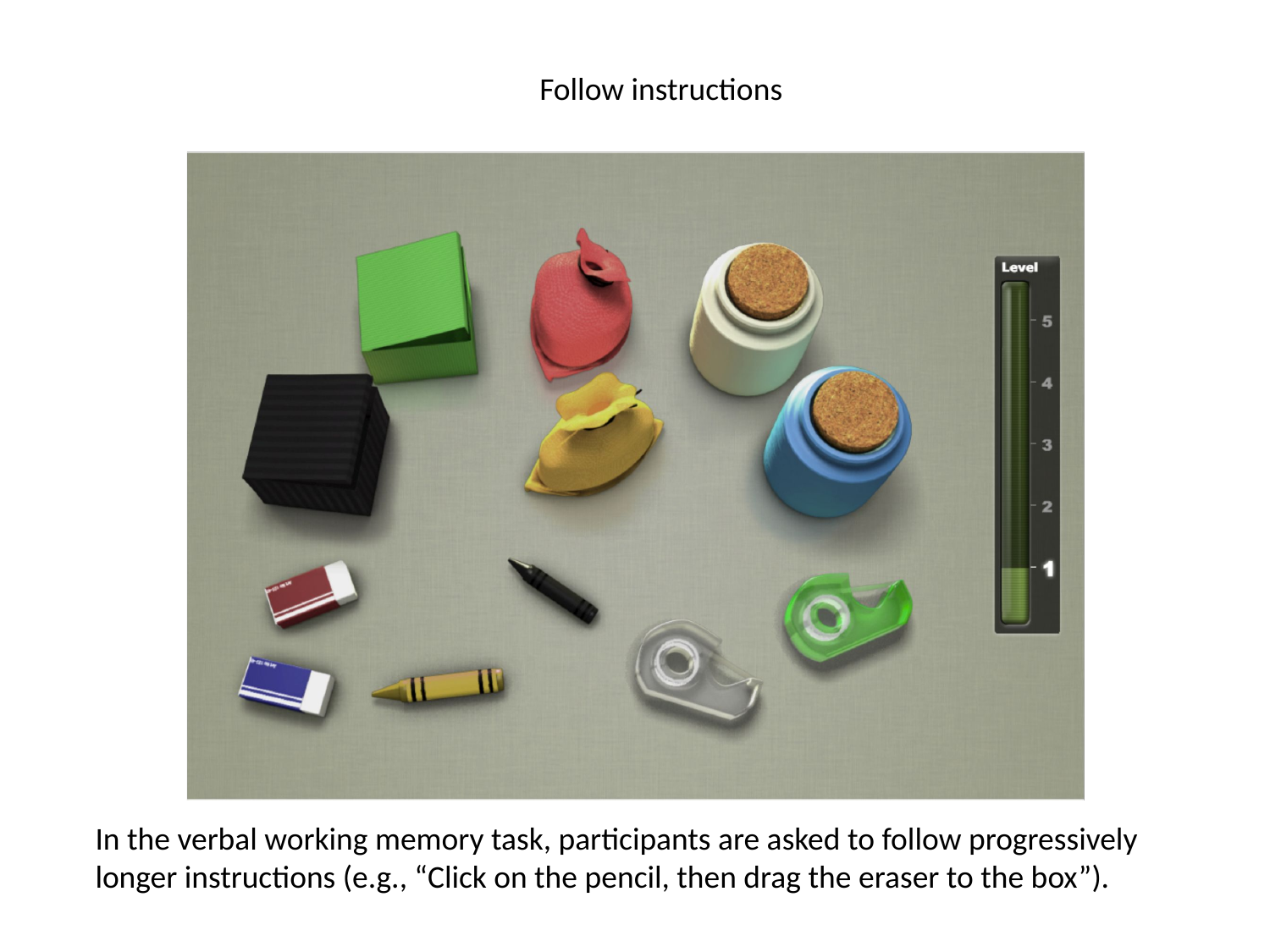

Follow instructions
In the verbal working memory task, participants are asked to follow progressively longer instructions (e.g., “Click on the pencil, then drag the eraser to the box”).

## Slide 4
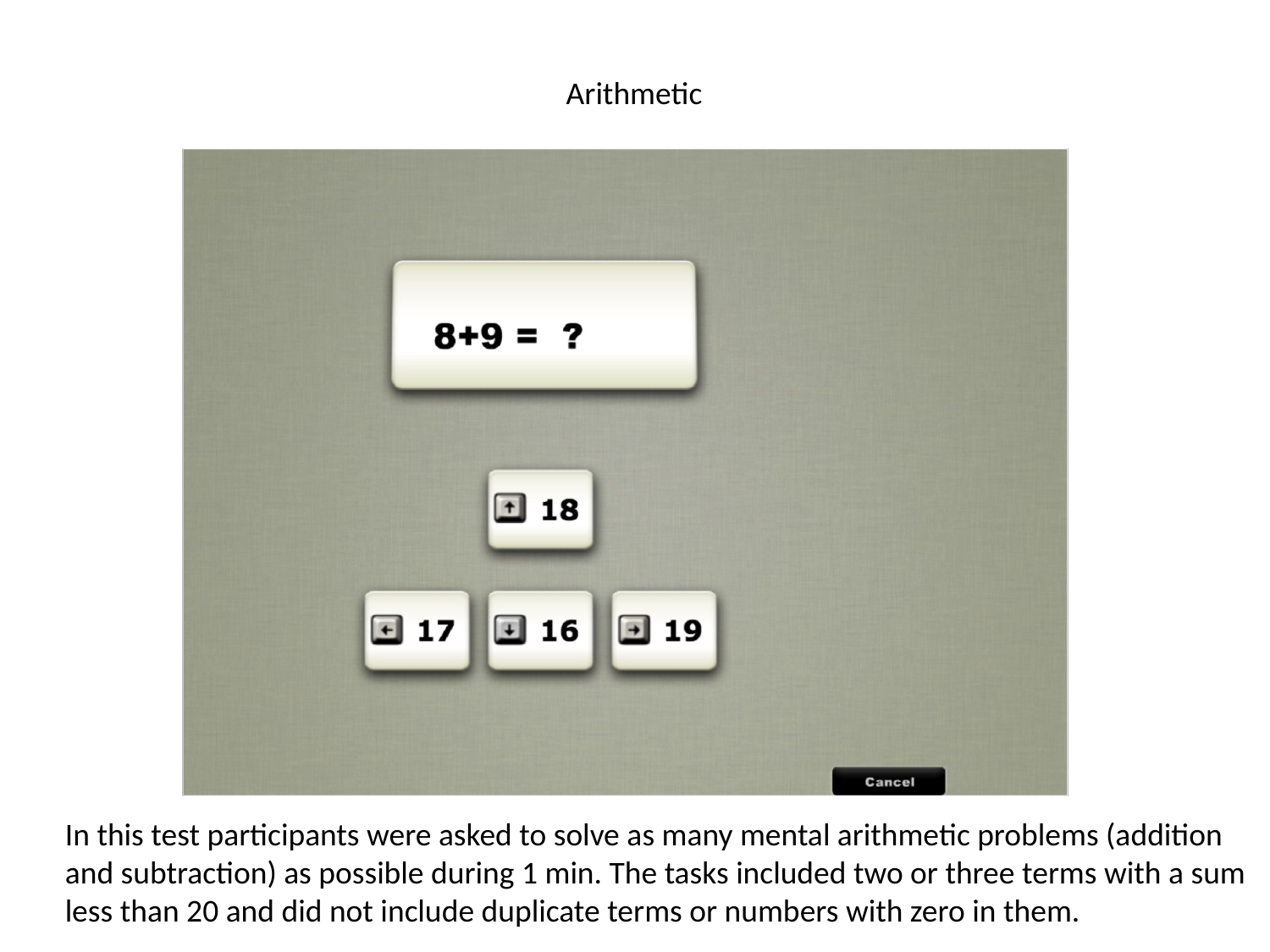

Arithmetic
In this test participants were asked to solve as many mental arithmetic problems (addition and subtraction) as possible during 1 min. The tasks included two or three terms with a sum
less than 20 and did not include duplicate terms or numbers with zero in them.
